# Systematic Evaluation of Light-Activatable Biohybrids for Anti-Glioma Photodynamic Therapy

**DOI:** 10.3390/jcm8091269

**Published:** 2019-08-21

**Authors:** Collin T. Inglut, Yan Baglo, Barry J. Liang, Yahya Cheema, Jillian Stabile, Graeme F. Woodworth, Huang-Chiao Huang

**Affiliations:** 1 Fischell Department of Bioengineering, University of Maryland, College Park, MD 20742, USA; 2 Department of Neurosurgery, University of Maryland School of Medicine, Baltimore, MD 21201, USA; 3 Marlene and Stewart Greenebaum Cancer Center, University of Maryland School of Medicine, Baltimore, MD 21201, USA

**Keywords:** photosensitizing biomolecule, photodynamic therapy, benzoporphyrin derivative, brain cancer, subcellular targeting

## Abstract

Photosensitizing biomolecules (PSBM) represent a new generation of light-absorbing compounds with improved optical and physicochemical properties for biomedical applications. Despite numerous advances in lipid-, polymer-, and protein-based PSBMs, their effective use requires a fundamental understanding of how macromolecular structure influences the physicochemical and biological properties of the photosensitizer. Here, we prepared and characterized three well-defined PSBMs based on a clinically used photosensitizer, benzoporphyrin derivative (BPD). The PSBMs include 16:0 lysophosphocholine-BPD (16:0 Lyso PC-BPD), distearoyl-phosphoethanolamine-polyethylene-glycol-BPD (DSPE-PEG-BPD), and anti-EGFR cetuximab-BPD (Cet-BPD). In two glioma cell lines, DSPE-PEG-BPD exhibited the highest singlet oxygen yield but was the least phototoxic due to low cellular uptake. The 16:0 Lyso PC-BPD was most efficient in promoting cellular uptake but redirected BPD’s subcellular localization from mitochondria to lysosomes. At 24 h after incubation, proteolyzed Cet-BPD was localized to mitochondria and effectively disrupted the mitochondrial membrane potential upon light activation. Our results revealed the variable trafficking and end effects of PSBMs, providing valuable insights into methods of PSBM evaluation, as well as strategies to select PSBMs based on subcellular targets and cytotoxic mechanisms. We demonstrated that biologically informed combinations of PSBMs to target lysosomes and mitochondria, concurrently, may lead to enhanced therapeutic effects against gliomas.

## 1. Introduction

Light-activatable biohybrid conjugates offer great promise for anti-cancer photodynamic therapy (PDT) due to their ability to improve pharmacokinetics, minimize clearance of photosensitizing agents, and enhance tumor-specific cytotoxic effects [1]. PDT is a light-based treatment modality that is mechanistically distinct from conventional chemotherapy, radiation, and thermal ablation [2,3,4]. Cytotoxicity related to PDT is governed by the localization of the photosensitizer, spatial confinement of near-infrared (NIR) light activation, and the production of highly reactive oxygen species (ROS; e.g., ^1^O_2_, H_2_O_2_, O_2_^•−^, ^•^OH) that confer toxicity to cellular and subcellular targets [2,3,4]. The direct damage to subcellular organelles (e.g., mitochondria, endoplasmic reticulum, and lysosome) allows for a more channeled activation of programmed cell death pathways (e.g., apoptosis, necroptosis, and autophagy), and therefore bypasses many other cell-death signaling pathways required for chemotherapy and radiation to be effective [2,3,4]. As a result, PDT has been shown to reverse chemoresistance, synergize with chemotherapeutics and biological agents, and overcome compensatory survival pathways used by numerous cancers to evade standard treatments [5,6,7,8,9,10].

In the past few decades, considerable attention has been given to improving the biocompatibility of organic photosensitizing agents, including porphyrins, chlorins, and synthetic dyes [11,12]. It is increasingly evident that photosensitizing biomolecules (PSBMs), based on chemical conjugation or association of photosensitizers with biomolecules, strongly influence the performance of a given photosensitizer in biological environments [1]. To harness the full potential of PSBMs for cancer treatment, it is of paramount importance to achieve a general and comprehensive understanding of how different types of biomolecules alter the uptake, subcellular localization, and cytotoxicity of photosensitizers. However, the numerous studies examining different PSBMs are not readily comparable as they typically cover a vast array of photosensitizers and treatment conditions [1]. This study systematically examines the impact of a panel of biomolecules on the behavior of benzoporphyrin derivative (BPD), a United States Food and Drug Administration (FDA)-approved photosensitizer now being routinely tested for cancer treatment and imaging [13,14,15].

Amongst the PSBMs that are currently available in the clinic, phospholipid-, polymer-, and antibody-based formulations are popular ways to improve the water solubility, pharmacokinetics, and biodistribution of photosensitizers; however, little is known about how these re-formulation strategies alter cellular uptake, subcellular localization, and the end effects in PDT regimes against glioblastoma. In 2000, phospholipid-associated BPD (i.e., Visudyne^®^) was approved by the FDA for PDT of wet age-related macular degeneration [13,14]. Accordingly, Visudyne^®^ is now being tested in several clinical trials evaluating PDT for pancreatic ductal adenocarcinoma (NCT03033225), [15] recurrent and metastatic prostate cancer (NCT03067051), metastatic breast cancer (NCT02939274), breast neoplasms (NCT02872064), and pleural malignancies (NCT02702700). In an early clinical study, Bown et al. tested the use of intravenous polyethylene glycol (PEG)-solubilized meso-tetrahydroxyphenyl chlorin (mTHPC) for enhanced PDT against locally advanced, unresectable pancreatic cancer [16]. In their study, light was delivered to the tumor percutaneously under computerized tomographic guidance, which achieved a 100% response rate and a median overall survival of 9.5 months. Later on, Moore et al. employed Cremophor^®^-emulsified palladium-bacteriopheophorbide (TOOKAD^®^) for successful vascular-targeted PDT against prostate cancer [17]. Regarding antibody-based PSBMs, Schmidt et al. were the first to conduct a clinical study using antibody-directed phthalocyanine conjugates for targeted PDT in patients with advanced ovarian carcinoma [18]. More recently, a PSBM-based system using a cetuximab-IR700 conjugate (RM-1929) was designed for photoimmunotherapy of head and neck cancer and showed encouraging results in a clinical trial (NCT02422979) [19]. While the primary motivation of re-formulated photosensitizers with PSBMs is to improve the photo- and physio-chemical properties of the agents in vivo, it is apparent that the complex interactions between cellular uptake, subcellular localization, and molecular targets are more likely to govern the observed phototoxicity of a given PSBM.

In this study, we prepared and characterized a series of PSBMs by conjugating BPD to (i) 1-palmitoyl-2-hydroxy-sn-glycero-3-phosphocholine (16:0 Lyso PC) phospholipid, (ii) distearoyl-phosphoethanolamine-polyethylene glycol (DSPE-PEG) phospholipid-polymer conjugate, or (iii) anti-epidermal growth factor receptor (EGFR) monoclonal antibody cetuximab (Cet). The study of lipid-conjugated tetraphenylporphyrin photosensitizers was first reported by Komatsu et al. in 2002 to form spherical unilamellar vesicles with a diameter of 100 nanometers (nm) [20]. This work was later followed by studies from Zheng, Lovell, and others using different linking strategies, photosensitizers, and lipids to synthesize porphysome nanovesicles for effective light-based therapy of tumors [21,22,23]. Studies from Soukous and Hamblin showed that the surface charge and PEGylation of polylysine-phytochlorin (Chlorin e6) conjugates alter tissue-localizing prosperities and play an important role related to the efficacy of PDT for ovarian cancer [24,25]. Hasan and others pioneered covalent conjugation of BPD to Cet for targeted photoimmunotherapy and fluorescence imaging [26,27,28]. The highly self-quenched BPD conjugated to Cet can be internalized by cancer cells through clathrin-mediated endocytosis and de-quenched via lysosomal proteolysis of the antibody [27,29]. Here, using 16:0 Lyso PC-BPD, DSPE-PEG-BPD, Cet-BPD, and free-form BPD, we present a full physical, photochemical, cellular uptake, and subcellular localization investigation under controlled experimental conditions. We explored how these properties relate to PSBM cytotoxicity in human glioblastoma cells.

## 2. Methods

### 2.1. PSBM Preparation and Purification

Three types of PSBMs were synthesized by covalently linking BPD (United States Pharmacopeia) to (i) 16:0 Lyso PC (MW. 495.63; Avanti-Polar Lipids), (ii) DSPE-PEG (MW. 2790.52; Avanti-Polar Lipids), or (iii) cetuximab (Cet) (MW. ∽152 KDa; Bristol-Myers Squibb). The 16:0 Lyso PC-BPD conjugate was prepared by crosslinking the carboxylic acid group of BPD to the hydroxyl functional group of 16:0 Lyso PC via the esterification reaction as described by others and us previously [23,30]. Briefly, 16:0 Lyso PC, BPD, 1-ethyl-3-(3-dimethylaminopropyl) carbodiimide (EDC), 4-(dimethylamino) pyridine (DMAP), and *N*,*N*-diisopropylethylamine (DIPEA) were mixed in dichloromethane at a fixed molar ratio of 1:5:50:25:60 (113 mg total) for 24 h at room temperature. Dichloromethane was removed via rotary evaporation, and the residue was suspended in 5 mL of methanol prior to Sephadex^®^ LH-20 gel chromatography column purification. Following which, methanol was removed via rotary evaporation and the purified 16:0 Lyso PC-BPD was stored at −20 °C. The DSPE-PEG-BPD conjugates were prepared using carbodiimide crosslinker chemistry as described by others previously [31]. Briefly, EDC, BPD, and DSPE-PEG were mixed in chloroform at a molar ratio of 50:10:1 (20 mg total) for 24 h at room temperature. Chloroform was removed via rotary evaporation, and the resulting thin film was hydrated with 1.5 mL of methanol prior to purification with a Sephadex^®^ LH-20 gel chromatography column. The purified DSPE-PEG-BPD conjugates were dried via rotary evaporation and then stored at −20 °C storage. Conjugation of BPD to Cet was achieved using carbodiimide crosslinker chemistry as described by our group and others [26,29]. Briefly, PEGylation of Cet was carried out by adding mPEG-NHS in DMSO (200 μL; 40,000 MW; 4 mg/mL) dropwise to Cet (2 mL; 2 mg/mL) and allowed to stir overnight at 400 rpm at room temperature. PEGylated Cet was reacted with the *N*-hydroxysuccinimidyl ester of BPD (BPD-NHS) at 1:9 molar ratios for 20 h. The resulting Cet-BPD conjugates were purified using a Zebra spin desalting column (7kDa MWCO; Thermo; pre-equilibrated with 30% DMSO in PBS), and then washed in a 20 mL centrifugal filter tube (30 kDa MWCO; Amicon) three times with 5% DMSO in PBS prior to storage at 4 °C. The purified 16:0 Lyso PC-BPD and DSPE-PEG-BPD conjugates were analyzed using matrix-assisted laser desorption ionization-time of flight mass spectrometry (MALDI-TOF MS; Bruker). The BPD concentration of PSBM was estimated using UV-Vis spectroscopy and the established molar extinction coefficient of BPD in DMSO (~34,895 M^−1^ cm^−1^ at 687 nm) [5]. The Cet-BPD purity was confirmed by gel fluorescence imaging analysis of free BPD following sodium dodecyl sulfate-polyacrylamide gel electrophoresis (SDS-PAGE). The protein content of Cet-BPD was evaluated by BCA protein assay (Thermo). BPD conjugation efficacy was determined by dividing the amount of BPD in the purified Cet-BPD by the total amount of BPD added initially.

### 2.2. Photophysical and Photochemical Characterizations

The fluorescence-based optical characterization (Ex/Em: 422/650–750 nm) of PSBMs in PBS or DMSO was determined using a multi-mode microplate reader (BioTek). Fluorescence photoactivity is defined as the maximal fluorescence intensity (FI) of PSBM in PBS divided by the maximal FI of PSBM in DMSO. The singlet oxygen yield of PSBM was studied in 96-well plates as described previously [5,29]. Briefly, BPD and singlet oxygen sensor green (SOSG) were fixed at 5 μM, and light (690 nm, 10 J/cm^2^, 10 mW/cm^2^) was delivered vertically. The microplate reader acquired the fluorescence signals of SOSG (Ex/Em: 504/525 nm).

### 2.3. Cell Culture

Human glioblastoma cell lines U251 and U87 were obtained from American Type Culture Collection (ATCC) and cultured in Eagle’s Minimum Essential Medium (EMEM) cell culture medium (Cellgro), supplemented with 10% (*v*/*v*) fetal bovine serum (Gibco), 100 U/mL penicillin and 100 μg/mL streptomycin (Lonza), according to the manufacturer’s instructions. The GBM39 patient-derived xenograft (PDX) was originally obtained from patients at the Mayo Clinic by Dr. Jann Sarkaria. Molecular genetic alterations and patient tumor information for GBM39 has been previously determined and described [32]. GBM39 was derived from the primary GBM tumor of a 51-year-old male prior to radiation, chemotherapy (Temozolomide), craniotomy, and Carmustine (BCNU) wafers (Gliadel). GBM39 cells were cultured in DMEM/F12 medium using StemPro NSC SFM Kit (ThermoFisher A1050901) including neural supplement, bFGF, and EGF, supplemented with 100 U/mL penicillin and 100 μg/mL streptomycin (Lonza), and 5 mM GltaMAX (GIBCO). GBM39 cells were cultured to 70% confluency in a Poly-l-lysine (Sigma) and laminin (GIBCO) coated culture flask before seeding. All cell lines were maintained in 5% CO_2_ at 37 °C. Cultures were tested mycoplasma free after resuscitation.

### 2.4. Photodynamic Therapy (PDT) of Glioblastoma Cells Using PSBMs

For PDT study, U251 and U87 cells were plated in 35-mm Petri dishes at a cell density of 7.5 × 10^4^ and 1.5 × 10^5^ cells per dish, respectively, to allow overnight culture. Attached cells were incubated in serum-free medium containing free BPD or PSBMs at desired concentrations for 24 h, at which time cells were irradiated with 690 nm red light (20 J/cm^2^, 150 mW/cm^2^, bottom illumination) in the fresh complete culture medium. Light activation was performed using the ML6600 series laser system (690 ± 5 nm; 1.5 W max output power; SMA fiber output with SMA905 connector, Modulight). Cell viability was determined by MTT assay at 24 h after 690 nm light irradiation. Cell survival fractions for PDT treated samples were normalized to the no treatment controls. The no treatment controls were incubated in serum-free medium with no exposure to free BPD or PSBM’s and were not subject to irradiation. Alternatively, cells were collected 4 h post-treatment and incubated with Annexin V-FITC and propidium iodide (Abcam, ab14085) for flow cytometry analysis (BD FACSCelesta). Mitochondrial membrane potential (ΔΨm) was examined via TMRE assay (Abcam). Briefly, U251 cells were cultured overnight in 96-well black wall plates at a density of 3000 cells/well, and then incubated with PSBM for 24 h (0.15 μM of free BPD, 0.25 μM of 16:0 Lyso PC-BPD, 0.5 μM of DSPE-PEG-BPD, and 0.5 μM of Cet-BPD). After incubation, cells were washed twice with PBS and exposed to 690 nm light (0 or 1 J/cm^2^, 10 mW/cm^2^). After PDT, cells were then incubated with TMRE (250 nM)-containing medium for 25 min, washed twice with 0.2% BSA within PBS, and subjected to fluorescence readings (Ex/Em: 549/575 nm) using a microplate reader (BioTek). Controls include no light irradiation and 150 μM of carbonilcyanide *p*-triflouromethoxyphenylhydrazone (FCCP). Lysosome damage was examined via LysoTracker Red incorporation. Briefly, cells were incubated with 0.15 μM free BPD, 0.25 μM 16:0 Lyso PC-BPD, 0.5 μM DSPE-PEG-BPD, or 0.5 μM Cet-BPD for 24 h and irradiated with 690 nm red light (20 J/cm^2^, 150 mW/cm^2^, bottom illumination). At 30 min after light treatment, cells were incubated with 100 nM LysoTracker Red in serum free medium for 15 min at 37 °C and washed twice with PBS. At least 20,000 events were collected by the flow cytometer (BD FACSCelesta) using the BD Horizon PE-CF594 filter (Ex/Em: 561/610) and then analyzed with FlowJo software.

### 2.5. PSBM Uptake and Localization Studies in Glioblastoma Cells

To evaluate PSBM uptake, U251 (or U87) cells were plated in 35-mm Petri dishes at 2 × 10^5^ and 2.5 × 10^5^ cells per dish, respectively. Attached cells with 80%–90% confluency were incubated in culture medium containing free BPD or PSBMs (0–1 μM, 1 mL) for 24 h. Cells were washed twice with PBS, harvested with trypsin, and lysed in Solvable solution (0.5 mL) at 4 °C. BPD fluorescence intensity (Ex/Em: 435/690 ± 20 nm) was measured using a microplate reader (BioTek) and converted to moles of BPD based on appropriate standard curves. Protein concentration was determined using the Pierce BCA Protein Assay. To verify PSBM uptake using fluorescence microscopy imaging (Lionheart, BioTek), cells were plated at the same density in a 24-well glass bottom plate, and incubated with 0.25 μM free BPD or PSBM for 24 h. Before imaging, cells were washed twice with PBS, and nuclei were stained with Hoechst nucleic acid stain. Live cell imaging was then performed to confirm aldehydes did not impact the analysis. Images were captured in quadruplet at specified locations for each well. Fluorescence intensity of BPD was quantified and normalized. Confocal fluorescence imaging studies were performed to determine the subcellular localization of PSBMs in glioblastoma cells. Briefly, cells were cultured overnight in 35-mm glass bottom dishes at 75,000 cells per dish and incubated with free BPD or PSBMs for 24 h before confocal imaging. After PBS washing, cells were stained with 1 mL 100 nM MitoTracker Green (M7514; Invitrogen) or 100 nM LysoTracker Red probes (DND-99; Invitrogen) for 30 min at 37 °C. Hoechst was utilized to stain the nuclei of the cells prior to confocal fluorescence imaging (Olympus FluoView-1000 confocal microscope) using a 10× or 40× objectives. Excitation of Hoechst/BPD, MitoTracker, and LysoTracker were carried out using 405 nm, 488 nm, and 559 nm lasers, respectively, with appropriate filters (Hoechst: 430–470 nm; BPD: 650–750 nm; MitoTracker: 500–540 nm; LysoTracker: 570–620 nm). Profile plots were created using ImageJ software to show co-localization of PSBM and mitochondria or lysosomes [33].

### 2.6. Immunoblotting

U251 cells (7.5 × 10^5^ cells per 35-mm Petri dish) were treated with PSBM for 24 h (0.15 μM of free BPD, 0.25 μM of 16:0 Lyso PC-BPD, 0.5 μM of DSPE-PEG-BPD, and 0.5 μM of Cet-BPD) followed by irradiated at 690 nm (1 J/cm^2^, 10 mW/cm^2^, bottom illumination) in the fresh culture medium. At 6 and 24 h post-PDT, cell lysates were collected in RIPA lysis buffer supplemented with 1% phosphatase and protease inhibitor cocktail. Protein lysates (20 µg) of each sample were separated on a NuPAGE 4–12% precast Bis-Tris gel (Mini-tank system, ThermoFisher) and transferred onto a polyvinylidene difluoride (PVDF) membrane. Subsequent to blocking with 5% BSA in TBST, the blot was incubated with primary antibody against LC3I/II (Cell Signaling, #4108) overnight at 4 °C followed by incubation with its secondary antibody for 1 h at room temperature based on the supplier’s advice. Visualization of protein bands was developed by chemiluminescence (SuperSignal, ThermoFisher) with exposure to Gel Imager (ProteinSimple). β-actin serviced as a loading control.

### 2.7. Statistical Analyses

Results are mean ± standard error of the mean (SEM). Statistical tests were carried out using GraphPad Prism. All experiments were carried out at least in triplicate. Specific tests and number of repeats are indicated in the figure captions. Reported *p* values are two-tailed. One-way ANOVA statistical tests and appropriate post hoc tests were carried out to avoid type one error. No exclusion criteria were used, and no data points were excluded from the analysis. Investigators were blinded to experimental groups wherever possible.

### 2.8. Ethical Approval and Informed Consent

All experimental protocols were approved by University of Maryland College Park, Department of Environmental Safety, Sustainability & Risk. The methods were carried out in accordance with the relevant guidelines and regulations.

## 3. Results and Discussion

### 3.1. Synthesis and Characterization of PSBMs

**Figure 1:**
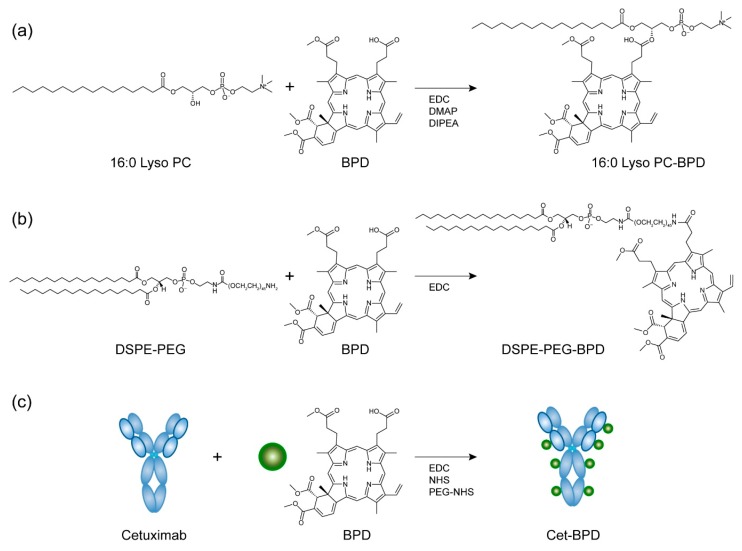
Synthesis reaction and chemical structure of photosensitizing biomolecules (PSBMs). (**a**) 1-palmitoyl-2-hydroxy-sn-glycero-3-phosphocholine (16:0 Lyso PC) was conjugated to benzoporphyrin derivative (BPD) via esterification reaction, resulting in a 1:1 ratio of 16:0 Lyso PC to BPD. Carbodiimide crosslinker chemistry was employed to conjugate BPD onto (**b**) distearoyl-phosphoethanolamine-polyethylene glycol (DSPE-PEG) and (**c**) monoclonal antibody cetuximab (Cet). Final conjugates contain 1:1 of DSPE-PEG-to-BPD and 1:7 ratio of Cet-to-BPD. Green circles represent BPD photosensitizers.

The 16:0 Lyso PC-BPD PSBMs were synthesized by an esterification reaction between the carboxyl groups of BPD photosensitizers and the alcohol groups of 16:0 Lyso PC lipids. The hydrophobic BPD placed along the alkyl side chain of 16:0 Lyso PC maintains an overall amphipathic structure of the 16:0 Lyso PC-BPD (Figure 1a). To prepare DSPE-PEG-BPD, BPD was coupled to the polar PEG-modified head group of DSPE-PEG via amide coupling, ultimately changing the amphipathic structure by capping the hydrophilic PEG head with a hydrophobic BPD molecule (Figure 1b). MALDI-TOF mass spectrometer analysis of the purified 16:0 Lyso PC-BPD conjugates showed a single primary product with a mass of 1196.4, compared to multiple products for the crude reaction mixture (Figure S1a). The observed peak mass for the purified DSPE-PEG-BPD PSBMs was 3602.1 (Figure S1b), which is similar to other’s findings [31]. The final yields of 16:0 Lyso PC-BPD and DSPE-PEG-BPD were approximately 20.1% ± 3.8% and 13.0% ± 3.7%, respectively. It has been well established that 16:0 Lyso PC-BPD and DSPE-PEG-BPD can self-assemble in aqueous medium into a variety of nano- to micron-scale vesicles when using the extrusion method [21,23,31]. The structure of the vesicles formed is dictated by the concentration of PSBMs as well as the self-assembling environment [21,23,31]. Here, our goal is to examine the cellular interaction of the monomeric PSBMs instead of their nano/micro-formulations. Under our experimental conditions, no nano- or micro-vesicles of 16:0 Lyso PC-BPD or DSPE-PEG-BPD were detected when using dynamic light scattering technique.

To prepare Cet-BPD conjugates (Figure 1c), 7 BPD photosensitizers were attached to the lysine groups of each anti-EGFR antibody Cet at a conjugation efficiency of 66.1% ± 3.6%. PEGylation of Cet before BPD conjugation was necessary to prevent the aggregation of Cet-BPD conjugates [29]. We have previously confirmed that conjugation of 6–7 BPD molecules per Cet does not impair the selective binding and internalization of Cet in EGFR positive cancer cells [29]. Sodium dodecyl sulfate-polyacrylamide gel electrophoresis (SDS-PAGE) was used to identify and monitor the purity of Cet-BPD, which is less than 1% free BPD (Figure S2). Similar to 16:0 Lyso PC-BPD and DSPE-PEG-BPD, Cet-BPD can be incorporated into nanoconstructs by linking them to the outer layer of nanoparticles to achieve targeting functionality [11,12,29,34,35].

### 3.2. Photoactivity of PSBMs

**Figure 2:**
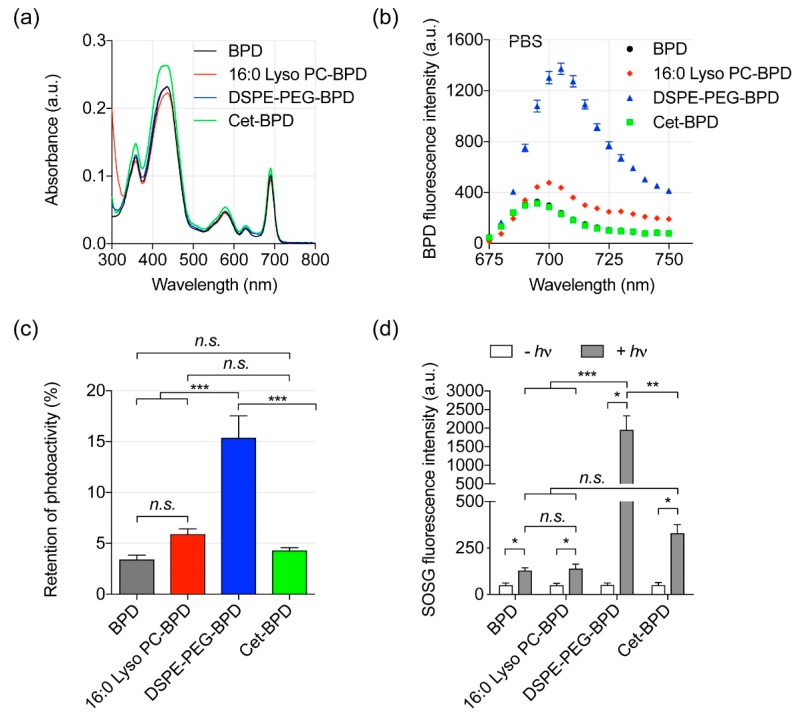
Photophysical and photochemical characterization of PSBMs. (**a**) Representative absorbance spectra of free BPD, 1-palmitoyl-2-hydroxy-sn-glycero-3-phosphocholine-BPD (16:0 Lyso PC-BPD), distearoyl-phosphoethanolamine-polyethylene glycol-BPD (DSPE-PEG-BPD), and cetuximab-BPD (Cet-BPD) in dimethyl sulfoxide (DMSO). Conjugation of BPD on biomolecules did not alter the Q-band (690 nm), which is used to activate BPD for photodynamic therapy (PDT). (**b**) Biomolecular conjugation of BPD on DSPE-PEG and 16:0 Lyso PC regained fluorescence intensity of BPD under physiological relevant condition. Fluorescence quenching was observed in Cet-BPD as in free BPD. (**c**) Photoactivity is defined as the maximal fluorescence intensity (FI) of PSBM in PBS divided by the maximal FI of PSBM in DMSO. Photoactivity of BPD was restored when BPD was conjugated onto DSPE-PEG compared to that of free BPD. Fluorescence quenching of BPD on Cet-BPD and 16:0 Lyso PC-BPD yield similar photoactivity compared to that of free BPD. (*n* = 6, *** *p* < 0.001, *n.s.*: nonsignificant, one-way ANOVA with Tukey’s post hoc test). (**d**) SOSG reports ^1^O_2_ production from photo-activated (690 nm, 10 J/cm^2^; 10 mW/cm^2^) PSBMs and free BPD in PBS at a fixed BPD concentration of 5 μM. The ^1^O_2_ production of DSPE-PEG-BPD is 4–20-fold higher than that of free BPD, Cet-BPD, and 16:0 Lyso PC-BPD. (*n* = 3, * *p* < 0.05, ** *p* < 0.01, *** *p* < 0.001, one-way ANOVA with Tukey’s post hoc test).

Conjugation of BPD to biomolecules is an expedient strategy to improve the photoactivity of BPD under biologically relevant conditions. In clinical PDT practice, systemic BPD photosensitizers are commonly activated by 690 nm light because NIR light penetrates deeper into the tissues than light of a shorter wavelength [15]. Here, we showed that the conjugation of BPD to 16:0 Lyso PC, DSPE-PEG, or Cet does not alter the Q band (690 nm) or the Soret peak (435 nm) of BPD (Figure 2a). A red shift of the Q band was not anticipated, as the lipid conjugation reaction does not reduce the number of double bonds in the pyrrole rings of BPD [36]. We further evaluated the fluorescence emission spectra (650–750 nm) of free BPD and PSBMs (i.e., 16:0 Lyso PC-BPD, DSPE-PEG-BPD, or Cet-BPD) in phosphate buffered saline (PBS) and dimethyl sulfoxide (DMSO), using an excitation wavelength of 435 nm. The aggregation of free BPD in PBS led to a nearly complete (>95%) fluorescence quenching as compared to fully solubilized BPD in DMSO (Figure 2b). DSPE-PEG-BPD restored the fluorescence emission signal of BPD molecules in PBS and was the most effective at retaining up to 15% photoactivity of the BPD (Figure 2c). In contrast, 16:0 Lyso PC-BPD demonstrated less retention of photoactivity compared to that of DSPE-PEG-BPD. This is likely caused by the improved stability due to the presence of PEG in DSPE. Cet-BPD showed the least retention of photoactivity at ~5%. This can be explained by our previous report and others that Cet-BPD conjugates at 1:7 ratio display up to 7-fold BPD static quenching [26,27]. More importantly, these highly self-quenched BPD molecules on Cet can be de-quenched by cancer cells upon lysosomal proteolysis of the Cet antibody. This unique ‘tumor-activation’ function of Cet-BPD can be used to improve the selective delivery of BPD for a safer photoimmunotherapy as shown by others and ourselves [27,29].

Type II photosensitization is the dominant process in BPD-based PDT and produces highly reactive singlet oxygen (^1^O_2_), which confers toxicity to nearby targets by oxidizing proteins and lipids [2]. In this study, ^1^O_2_ generation from light-activated PSBMs or free BPD was monitored indirectly by using the fluorescence probe singlet oxygen sensor green (SOSG). We have previously confirmed that the BPD fluorescence is negligible at the wavelength used to monitor the SOSG fluorescence [5]. Upon 690 nm light activation, the SOSG fluorescence intensity generated by DSPE-PEG-BPD was at least 4-fold higher than that generated by free BPD, Cet-BPD, and 16:0 Lyso PC-BPD (Figure 2d). This correlates with our finding of DSPE-PEG-BPD exhibiting the highest retention of photoactivity (Figure 2c). The SOSG fluorescence intensity generated from Cet-BPD was not significantly higher than that produced by free BPD and 16:0 Lyso PC-BPD upon light activation (*p* > 0.05, Figure 2d). This is likely due to the fluorescence quenching shown in Figure 2c. This data suggests that DSPE-PEG-BPD, with relatively stronger fluorescence emission spectra, improves the ^1^O_2_ production under physiologically relevant conditions during light activation, compared to free BPD, Cet-BPD, and 16:0 Lyso PC-BPD; which may lead to a higher phototoxicity response for PDT.

### 3.3. Near-Infrared (NIR) Light-Mediated Photodestruction of Glioblastoma Cells Using PSBMs

**Figure 3:**
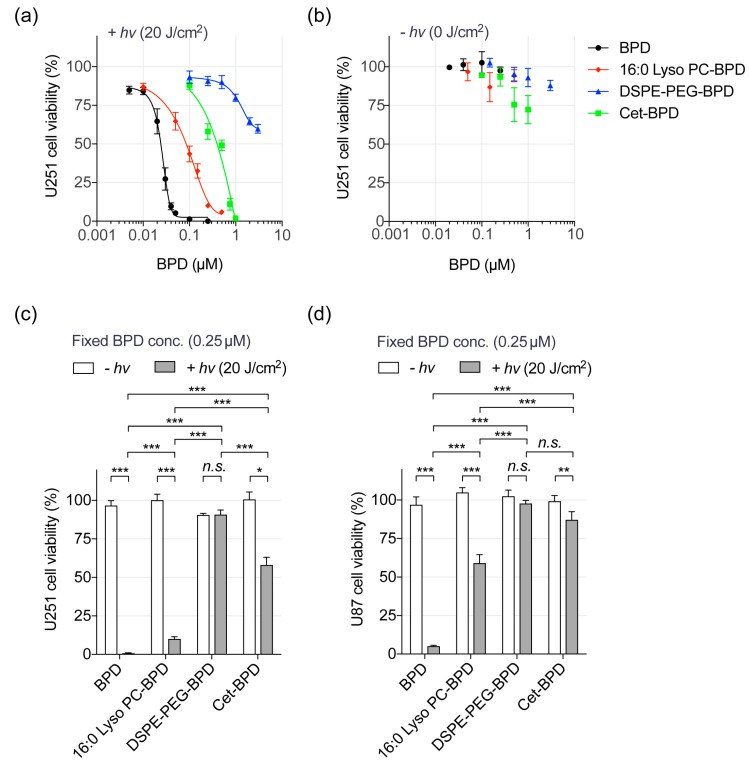
Near-infrared (NIR) light activation of PSBMs leads to the phototoxicity of glioblastoma cells. (**a**) U251 glioblastoma cells were incubated with PSBMs at different BPD concentrations (0–3 µM) for 24 h prior to light illumination (0 or 20 J/cm^2^, 150 mW/cm^2^). Cell viability was evaluated via MTT assay at 24 h after light activation. Free BPD exhibited the highest phototoxicity with IC_50_ of 0.44 µM × J/cm^2^, while 16:0 Lyso PC-BPD, Cet-BPD, and DSPE-PEG-BPD showed IC_50_ of 1.68 µM × J/cm^2^, 8.0 µM × J/cm^2^, and >60 µM × J/cm^2^, respectively. Light-activated PSBMs (+*hv*) resulted in a significant loss of U251 viability compared to (**b**) PSBM treatments without light activation (–*hv*). (**c**) U251 and (**d**) U87 glioblastoma cells were incubated with (i) free BPD, (ii) 16:0 Lyso PC-BPD, (iii) DSPE-PEG-BPD, or (iv) Cet-BPD at 37 °C for 24 h prior to light irradiation at a fixed PDT dose of 5.0 µM × J/cm^2^ (i.e., 0.25 µM BPD and 20 J/cm^2^). Free BPD induced the highest phototoxicity among the PSBMs at 24 h after light activation. (*n* > 3, * *p* < 0.05, ** *p* < 0.01, *** *p* < 0.001, two-tailed *t*-test).

In the above studies, we have shown that PSBMs could improve the photoactivity of BPD. We next investigated if this improvement in photoactivity translates to an enhancement in phototoxicity in vitro using two EGFR-positive glioblastoma cell lines (U251, U87) (Figure 3). Here, 16:0 Lyso PC-BPD, DSPE-PEG-BPD, Cet-BPD, and free BPD were incubated with glioblastoma cells at different concentrations (0–3 μM) for 24 h prior to 690 nm light activation (20 J/cm^2^, 150 mW/cm^2^, Figure 3a). The 16:0 Lyso PC-BPD, DSPE-PEG-BPD, and free BPD all showed negligible dark toxicity in U251 or U87 cells (Figure 3b–d). It is not surprising that Cet-BPD (above 0.5 μM) showed a modest 25% dark toxicity in U251 cells (Figure 3b), as it has been verified that the conjugation of seven BPD molecules onto Cet does not take away the targeting and therapeutic functionality of Cet [27,29]. At 24 h following PDT, the viability of cancer cells was assessed by MTT assay. Free BPD was found to be the most potent PDT agent with an IC_50_ of 0.44 μM × J/cm^2^ in U251 cells (Figure 3a), despite having the least retention of photoactivity and the lowest singlet oxygen yield under physiological conditions (Figure 2c,d). A significant decrease in phototoxicity was observed with 16:0 Lyso PC-BPD (IC_50_: 1.68 μM × J/cm^2^), Cet-BPD (IC_50_: 8 μM × J/cm^2^), and DSPE-PEG-BPD (IC_50_: >60 μM × J/cm^2^) (Figure 3a,c). This remarkable variation in the photo-cytotoxicity of PSBMs was also observed in the U87 cell line (Figure 3d). At a fixed PDT dose of 5 μM × J/cm^2^, free BPD reduced the viability of U87 cells by over 95%, and the loss of cell viability was dramatically reduced to approximately 40%, 13%, and 2% when using 16:0 Lyso PC-BPD, Cet-BPD, and DSPE-PEG-BPD, respectively (Figure 3d). This difference in PDT efficacy does not correlate with the photoactivity and singlet oxygen yields of PSBMs in physiologically relevant buffers. We showed that DSPE-PEG-BPD is the most photoactive formulation in a cell-free system, but it has the poorest in vitro PDT efficacy against glioblastoma cells. These results motivated us to evaluate the cellular uptake and location of PSBMs.

### 3.4. Extraction Method and Fluorescence Imaging to Quantify PSBM Uptake

**Figure 4:**
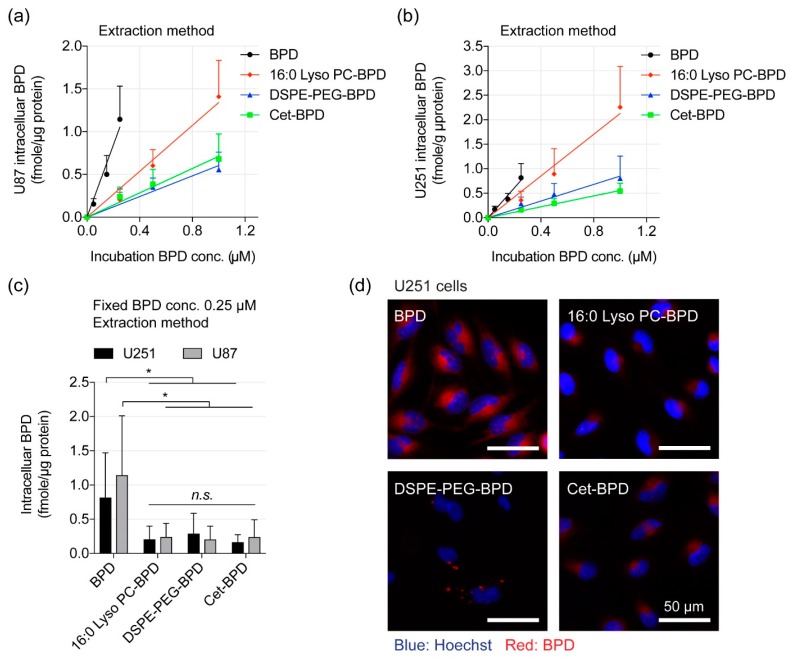
Cellular uptake of PSBMs and free BPD. Quantification of (**a**) U87 and (**b**) U251 intracellular BPD concentrations at 24 h post-incubation with free BPD, 16:0 Lyso PC-BPD, DESP-PEG-BPD, and Cet-BPD (0–1 µM) using extraction method. (**c**) Comparison of uptake of PSBM and free BPD at 0.25 µM in both U87 and U251 cell lines. (*n* = 5, * *p* < 0.05). (**d**) Representative fluorescence imaging of BPD photosensitizer (red) in U251 cells incubated with 0.25 µM free BPD or PSBMs for 24 h. Nuclear staining (blue, Hoechst); scale bar = 50 µm.

We compared the uptake of free BPD and PSBMs in U251 and U87 cells at different drug incubation concentrations (0–1 μM). After 24 h of incubation in U87 cells, free BPD showed the highest percentage of cellular uptake at 0.36% ± 0.08% per μg of protein, compared to 16:0 Lyso PC-BPD uptake at 0.13% ± 0.02% per μg of protein. Both DSPE-PEG-BPD and Cet-BPD had the lowest uptake percentage in U87 cells at 0.06% ± 0.01% per μg of protein and 0.07% ± 0.0134% per μg of protein, respectively (Figure 4a). Similarly, the reduced cellular uptake of PSBMs was observed in U251 cells (Figure 4b). Comparative analyses showed comparable PSBM uptake between the U87 and U251 cells at the concentrations used (Figure S3). It is likely that the stealth function of PEG, or the overall change in amphipathic structure, inhibited the cellular uptake of DSPE-PEG-BPD [37]. Additionally, we have also reported that the limited intracellular uptake of Cet-BPD is due to the low photosensitizers-to-antibody ratio of Cet-BPD required for maintaining the antibody’s targeting ability, as well as the limited amount of EGFR molecules presented on the cancer cell surface [29]. The cellular uptake of BPD (or PSBM) was further correlated with PDT-mediated viability reduction to determine the PDT efficiency (i.e., percent of cell viability reduction per fmole of intracellular BPD). Interestingly, the PDT efficiency of 16:0 Lyso PC-BPD (~2.2%) was found to be significantly higher than that of free BPD (~1.1%), DSPE-PEG-BPD (0.1–0.3%), and Cet-BPD (0.6–0.9%) in both U87 and U251 cell lines (Table 1). These results suggest that the cytotoxic mechanisms of PSBM-PDT are different than those of BPD-PDT.

Light activation of photosensitizers not only result in cytotoxicity but can also generate fluorescence signal from the relaxation of excited singlet-state photosensitizers back to the ground state. Recently, Gliolan^®^ (aminolevulinic acid hydrochloride, ALA HCL) has been approved for use to visualize malignant tissue during glioma surgery [38]. This use of ALA-induced fluorescent protoporphyrin IX (PpIX) to accurately define brain tumor margins is critical to achieving optimal surgical outcomes [39]. We next tested if PSBMs at 0.25 μM can be used for fluorescence imaging of U251 cancer cells (Figure 4c). After 24 h incubation at 37 °C and followed by washing to remove loosely attached or non-attached agents, live cells treated with free BPD showed the highest BPD fluorescence signal, compared to cells treated with 16:0 Lyso PC-BPD, DSPE-PEG-BPD, or Cet-BPD (Figure 4c). Quantification of the fluorescence images showed that free BPD exhibited ~2–10 fold higher fluorescence signal in U251 cells, compared to using 16:0 Lyso PC-BPD, DSPE-PEG-BPD, or Cet-BPD (Figure S4). While it is clear that free BPD uptake is greater than that of the PSBMs, extraction data reveals that uptake is not significantly different for 16:0 Lyso PC-BPD, DSPE-PEG-BPD, and Cet-BPD at the fixed incubation concentration of 0.25 μM. However, fluorescent imaging quantification shows 16:0 Lyso PC-BPD is slightly higher than DSPE-PEG-BPD and Cet-BPD. It is well established that fluorescent probes can respond to changes in their microenvironments (e.g., pH), [40,41] which possibly explains this observation. While fluorescent imaging is warranted, the more accurate method is extraction due to BPD being solubilized under the same conditions. It is obvious that our ability to effectively image cancer cells correlates with the amount of the photosensitizing agents associated with the cells. However, this data does not suggest that 16:0 Lyso PC-BPD, Cet-BPD, and DSPE-PEG-BPD are poor imaging agents, as the signal-to-background ratio of tumor to the surrounding normal tissue is a more important parameter to be carefully investigated in vivo.

### 3.5. Altered Subcellular Localization PSBM Leads to a New Combination PDT Approach for Glioblastoma.

**Figure 5:**
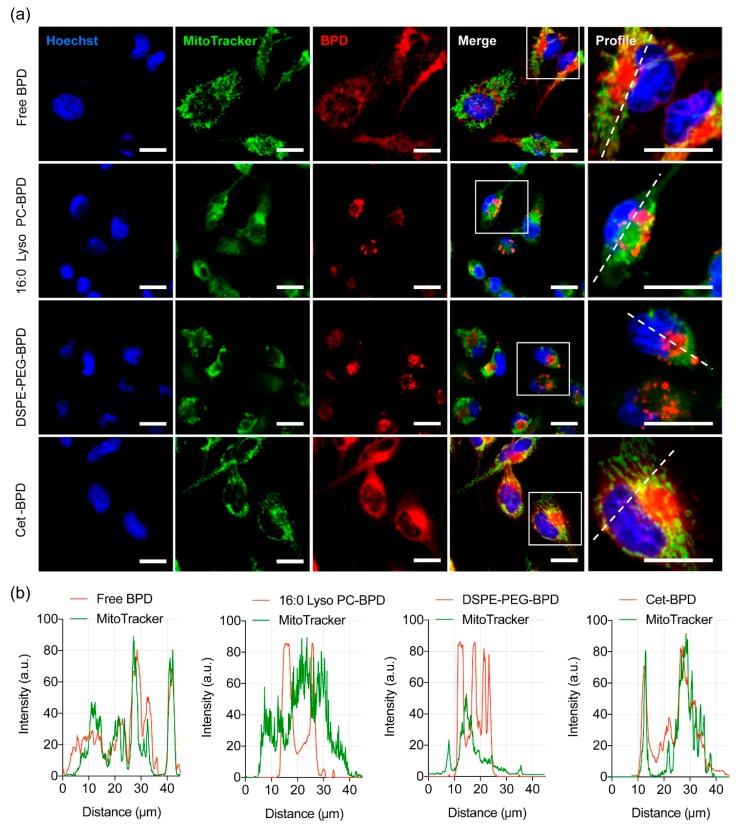
Mitochondrial localization of free BPD and PSBMs in U251 cells. (**a**) Representative confocal fluorescence images of U251 cells obtained after 24 h of incubation with free BPD, 16:0 Lyso PC-BPD, DSPE-PEG-BPD, and Cet-BPD. BPD signal pseudo-colored in red. Mitochondria were stained with 100 nM MitoTracker (M7514; pseudo-colored in green); merging of images where yellow indicates co-localization of BPD (red) and mitochondria (green). Nuclear staining (Hoechst; pseudo-colored in blue). Scale bar = 20 µm. (**b**) Profile plots were created using ImageJ software showing co-localization of mitochondria and free BPD (or PSBM) [33].

**Figure 6:**
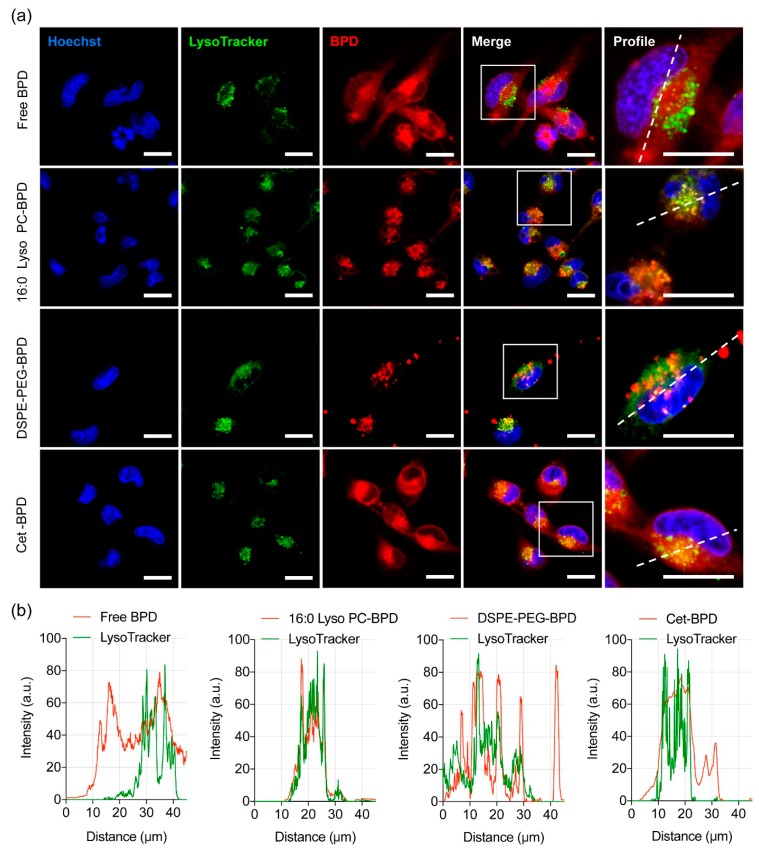
Lysosomal localization of free BPD and PSBMs in U251 cells. (**a**) Representative confocal fluorescence images of U251 cells obtained after 24 h of incubation with free BPD, 16:0 Lyso PC-BPD, DSPE-PEG-BPD, and Cet-BPD. BPD signal pseudo-colored in red. Lysosomes were stained with 100 nM of LysoTracker probes (DND-99; pseudo-colored in green); merging of images where yellow indicates co-localization of BPD (red) and lysosomes (green). Nuclear staining (Hoechst; pseudo-colored in blue). Scale bar = 20 µm. (**b**) Profile plots were created using ImageJ software showing co-localization of lysosome and free BPD (or PSBM) [33].

One of the preferred sites of localization for BPD photosensitizer is mitochondria [42,43]. Activation of BPD by light causes the photodynamic disruption of the mitochondrial membrane, which triggers the release of cytochrome c, a potent initiator of apoptotic cell death. PDT has also been shown to photochemically degrade the anti-apoptotic protein Bcl-2, while leaving pro-apoptotic proteins Bad and Bax unaffected [42,43]. This shifts the balance in the target cells from an anti-apoptotic state to a more pro-apoptotic state. To determine whether PSBMs are localized to the mitochondria, we incubated U251 cells with PSBMs for 24 h and treated cells with MitoTracker prior to examination using a confocal fluorescence microscope (Figure 5a). U251 cells that had been incubated with free BPD and Cet-BPD exhibited a strong and diffuse fluorescence signal throughout the cells. Converging the BPD image and MitoTracker image gave a pseudo-colored yellow fluorescent pattern (Figure 5a), and the profile plot analyses confirmed the localization of free BPD and Cet-BPD at mitochondria (Figure 5b). In contrast, 16:0 Lyso PC-BPD clustered in the peri-nuclear region with a reduced degree of mitochondria localization in U251 cells (Figure 5). The DSPE-PEG-BPD fluorescence signal in U251 cells was minimal and heterogeneously distributed across different cells (Figure 5). It was further confirmed that the peri-nuclear staining of 16:0 Lyso PC-BPD was related more to lysosomal incorporation (Figure 6), compared to mitochondria, a trend similar to that observed by others [21].

**Figure 7:**
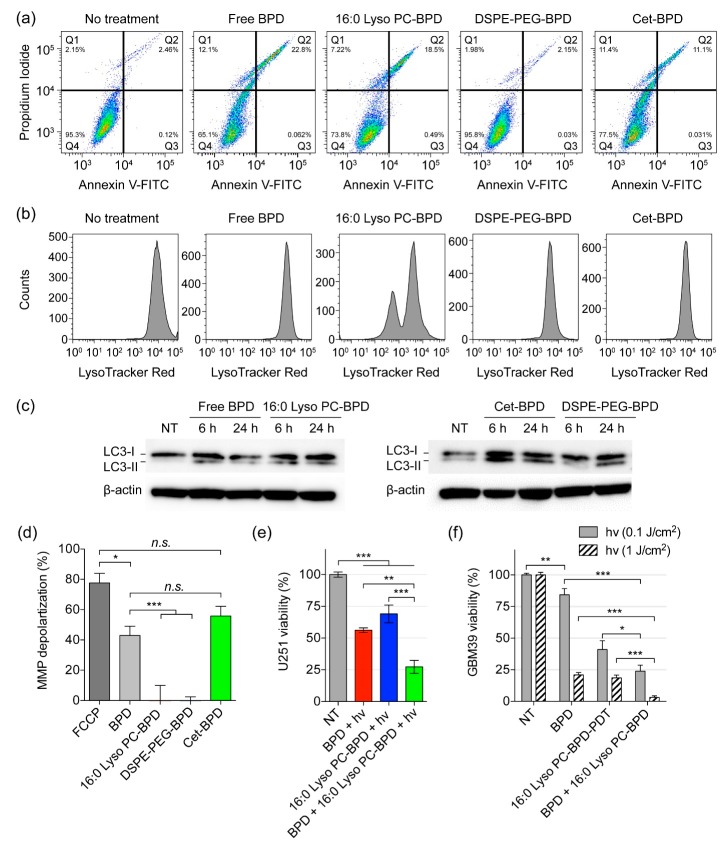
Acute cellular injury, lysosomal membrane permeabilization, autophagosome formation, mitochondrial membrane depolarization, and combination treatment using PSBMs and free BPD in U251 cells. Prior to PDT, cells were incubated with PSBMs at different concentrations for 24 h (i.e., 0.15 μM of free BPD, 0.25 μM of 16:0 Lyso PC-BPD, 0.5 μM of DSPE-PEG-BPD, and 0.5 μM of Cet-BPD) to achieve an equivalent intracellular BPD concentration (~0.35 μmoles per gram of protein). (**a**) Flow cytometric detection of apoptosis with Annexin V-FITC and propidium iodide was performed at 4 h after light irradiation (690 nm, 20 J/cm^2^, 150 mW/cm^2^) to detect living (Q4), early apoptotic (Q3), late apoptotic (Q2), and dead cells (Q1). Free BPD, 16:0 Lyso PC-BPD, and Cet-BPD all induced significant levels of apoptotic and dead cells, while DSPE-PEG-BPD had minor impact on cell viability. (**b**) Lysosomal membrane permeabilization was assessed 30 min after PDT. 16:0 Lyso PC-BPD induced the highest degree of lysosome damage, followed next by DSPE-PEG-PEG, free-BPD, and Cet-BPD. (**c**) Immunoblotting of LC3-II was assessed 6 and 24 h after PDT. All cells irradiated with light experienced an increase in LC3-II expression, indicating an accumulation of autophagosomes. (**d**) Mitochondrial membrane depolarization was assessed immediately after PDT. Free BPD and Cet-BPD induced the highest membrane depolarization compared to those of 16:0 Lyso PC-BPD and DSPE-PEG-BPD due to their localization in the mitochondria. (**e**,**f**) PDT using both free BPD and 16:0 Lyso PC-BPD led to a significant decrease in (**e**) U251 cell and (**f**) GBM39 viability compared to using free BPD or 16:0 Lyso PC-BPD alone. Cell viability was determined via MTT assay at 24 h post-PDT (690 nm, 0.1 J/cm^2^ for U251; 0.1 and 1 J/cm^2^ for GBM39, 10 mW/cm^2^, bottom illumination). (*n* > 3, * *p* < 0.05, ** *p* < 0.01, *** *p* < 0.001, *n.s.*: nonsignificant, one-way ANOVA with Tukey’s post hoc test).

We next investigate the ability of PSBM-based PDT to trigger apoptotic cell death, damage lysosomes, regulate autophagosome formation, and depolarize mitochondrial membrane potential (ΔΨm). To account for the variations in PSBM uptake, U251 cells were incubated with different concentrations of free BPD (0.15 μM), 16:0 Lyso PC-BPD (0.25 μM), DSPE-PEG-BPD (0.50 μM), or Cet-BPD (0.50 μM) to achieve an equivalent intracellular BPD concentration (~0.35 μmoles per gram of protein) prior to light irradiation. In order to compare the degree of which the PSBMs can induce apoptosis, flow cytometric detection of apoptosis with Annexin V-FITC and propidium iodide was performed. Light fluence was fixed at 20 J/cm^2^, which has been shown to reduce cell viability to nearly 0% at 24 h after treatment (Figure 3; MTT assay). For flow cytometry-based apoptosis detection, cells were examined at 4 h after light exposure to evaluate acute cellular injury (Figure 7a). Free BPD, 16:0 Lyso PC-BPD, and Cet-BPD all induced similar levels of apoptotic (~20%) and dead cells (~11%) (Figure 7a; quantification in Figure S5), while DSPE-PEG-BPD had little impact on cell viability (~96%) although having the same intracellular concentration. Due to lysosomal localization, PDT using 16:0 Lyso PC-BPD induced a high degree of lysosomal membrane permeabilization in U251 cells, decreasing the mean fluorescent intensity (MFI) of LysoTracker to 40% compared to that of the no treatment control (Figure 7b; MFI quantification in Figure S6). PDT treatment with free BPD, DSPE-PEG-BPD, and Cet-BPD modestly reduced the MFI of LysoTracker (Figure 7b; Figure S6), suggesting minimal lysosomal damage. A number of studies have already shown that PDT-mediated lysosomal damage could induce autophagy [44,45,46,47]. Here, we evaluated autophagosome formation at 6 and 24 h after PDT using free BPD or PSBMs. Immunoblotting results indicated that PDT using free BPD and PSBMs all induced an increase of LC3-II in comparison to the no treatment control at 24 h post-treatment (Figure 7c). Increase in LC3-II indicates the accumulation of autophagosomes, which can be seen even at 6 h after irradiation for free BPD, 16:0 Lyso PC-BPD, and Cet-BPD. However, this does not guarantee autophagic degradation [48]. Due to mitochondrial localization (Figure 5), PDT using free BPD or Cet-BPD induced a high level of ΔΨm depolarization in U251 cells (Figure 7d). No ΔΨm depolarization was observed using 16:0 Lyso PC-BPD and DSPE-PEG-BPD for PDT of U251 cells (Figure 7d). A potent mitochondrial oxidative phosphorylation uncoupler, *p*-triflouromethoxyphenylhydrazone (FCCP), was used as a positive control for ΔΨm depolarization [49]. Considering the time of incubation used (i.e., 24 h) in our study and above findings, 16:0 Lyso PC-BPD co-localization with MitoTracker (Figure 6b) could potentially be associated with auto-lysosomes containing mitochondria as suggested in Martins’ study [50]. The observed Cet-BPD and free BPD co-localization with LysoTracker could also be associated with auto-lysosomes containing mitochondria (Figure 6b). Further investigations into the cell/patient line-dependent phototoxicity and autophagy-associated cell death of PSBMs are warranted.

Studies by Martins et al., Coincotta et al., and Kessel showed that PDT directed against both lysosomes and mitochondria could enhance the overall efficacy of photo-killing of malignant human cells, murine hepatoma cells, and sarcoma [30,50,51,52,53,54]. Some of the earlier studies utilized different types of photosensitizers for lysosomal targeting (e.g., NPe6, EtNBs) and mitochondrial targeting (e.g., BPD), and thus require different wavelengths of light for PDT activation (i.e., NPe6, λ_max_ at 630 nm; EtNBs, λ_max_ at 652 nm; BPD λ_max_ at 690 nm). While PDT with photosensitizers accumulating in mitochondria leads to the loss of ΔΨm and the release of pro-apoptotic mediators (e.g., cytochrome c), light activation of photosensitizers that localizes in lysosomes could induce cell death via the release of proteolytic lysosomal enzymes [55]. Rizvi et al. have shown that 690 nm light action of Visudyne^®^ and lipid-anchored liposomal BPD enhances PDT outcomes via multi-organelle targeting [30,54]. Similarly, here, we showed that single wavelength (i.e., 690 nm) light activation of both free BPD and 16:0 Lyso PC-BPD, which target mitochondria and lysosomes, respectively, could achieve a significant reduction in viability at 72.6% ± 5.1%, compared to the modest viability reduction achieved by BPD-PDT (43.8% ± 1.8%) or 16:0 Lyso PC-BPD-PDT (30.9% ± 6.9%) (Figure 7e). Similarly, PDT using a mixture of BPD and 16:0 Lyso PC-BPD significantly reduced the viability of GBM39 cells by ∽80%, compared to that using free BPD (15.7% ± 11.9% viability reduction) or 16:0 Lyso PC-BPD (58.9% ± 6.9% viability reduction) alone (Figure 7f). In contrast to that observed in U87 and U251 cell lines (Figure 3), 16:0 Lyso PC-BPD was found to be more phototoxic compared to free BPD in GBM39 cells at a light fluence of 0.1 J/cm^2^ (Figure 7f). Thus, further mechanistic investigations into the cell/patient line-dependent phototoxicity of PSBMs are needed. We speculate that GBM39 cells showed resistance to the free BPD could be due to the activation of pro-survival mitophagy to maintain cell homeostasis. Thus, by compromising lysosomal function, as done using 16:0 Lyso PC-BPD, GBM39 cells become more sensitive to photodamage triggered by BPD-PDT. Martins and others have already showed this concept [50]. At a higher fluence of 1.0 J/cm^2^, both 16:0 Lyso PC-BPD and free BPD led to GBM39 viability reduction of approximately 80%, while the combination reduced virtually 100% of the GBM39 cell viability. While the combinations of free BPD and 16:0 Lyso PC-BPD led to a greater decrease in cell viability compared to monotherapy, a synergistic effect (Two-way ANOVA, interaction *p* value < 0.0001, GraphPad Prism) was only seen within the GBM39 cells at 1.0 J/cm^2^. A follow up full dose–response matrix study is required to identify and quantify synergistic, additive, and antagonistic dose regions. Additional animal research is also warranted to examine the benefits of combining 16:0 Lyso PC-BPD and free BPD to enhance the overall PDT efficacy.

## 4. Conclusions

In this study, we synthesized a panel of BPD-based PSBMs with different molecular weights and physiochemical properties. We systematically analyzed how different classes of biomolecules (i.e., lipid-, PEG-, and antibody-based) influence the physical, photochemical, and biological properties of the BPD photosensitizers. The simple and reproducible formulation strategies did not alter the Q-band of BPD for PDT using 690 nm light activation. The instability of free BPD in physiologically relevant media led to a complete fluorescence quenching. Conjugation of BPD to 16:0 Lyso PC or DSPE-PEG slightly improved BPD’s fluorescence emission by up to 20%. Static quenching of BPD molecules on cetuximab was expected and could be de-quenched upon lysosomal proteolysis of Cet-BPD for PDT, confirming previous findings [27,29]. This distinct property of Cet-BPD offers a unique opportunity to spatiotemporally target EGFR expressing cancer cells for selective PDT [27,29]. For all PSBMs and free BPD, a strong correlation between the degree of photoactivity and singlet oxygen yield was observed in a cell-free system. However, no correlation was found between the evaluated physicochemical properties and the photodynamic therapeutic index of PSBMs in two glioblastoma cell lines (U87, U251).

The uptake kinetics and subcellular localization of PSBMs were found to be the more critical factors for effective PDT and fluorescence imaging. Despite lower singlet oxygen production, the 16:0 Lyso PC-BPD exhibited enhanced delivery of BPD into glioblastoma cells, compared to DSPE-PEG and Cet-BPD, and could be visualized by fluorescence imaging. While the clathrin-mediated endocytosis pathway and lysosomal proteolysis mechanism of Cet-BPD in cancer cells have been well-established by us and others, here, we present the new evidence that proteolyzed Cet-BPD co-localizes to mitochondria to efficiently induce mitochondrial membrane (ΔΨm) depolarization upon light activation in glioblastoma cells. Light activation of 16:0 Lyso PC-BPD or DSPE-PEG-BPD did not induce ΔΨm depolarization. This can be explained by the localization of 16:0 Lyso PC-BPD in lysosomes, as well as the heterogeneous and negligible uptake of DSPE-PEG-BPD in glioblastoma cells. Our findings suggest that engineering PSBM for effective PDT is enabled by close examination of cellular trafficking and subcellular localization in order to predict their effectiveness. Importantly, it is apparent that no single parameter is predictive. Compared to PSBMs, free BPD was found to be a more potent PDT agent in vitro due to its higher cellular uptake and mitochondrial incorporation, which leads to a pronounced mitochondrial oxidative stress with apoptosis activation.

It is well accepted that the subcellular localization of the photosensitizer plays a significant role in the type of cell death mechanism that predominates. Employing different types of photosensitizers or functionalizing photosensitizers with targeting moieties to target one or multiple subcellular organelles (e.g., mitochondria, lysosome, endoplasmic reticulum, golgi, plasma membrane) have also been reported [51,52]. Here, we verified that 16:0 Lyso PC redirects BPD to lysosomes. More importantly, we demonstrated that single wavelength light activation of both free BPD and 16:0 Lyso PC-BPD targeting multiple subcellular organelles is a superior approach compared to using free BPD or 16:0 Lyso PC-BPD alone. Above insights of the molecular and cytotoxic mechanisms of PSBMs are critical to inform the design, prediction, and evaluation of more sophisticated photo-nanomedicine for glioma PDT and imaging.

In summary, our results reveal the variable trafficking and cytotoxicity of clinically relevant PSBMs for PDT, providing important insights into methods of PSBM evaluation as well as strategies to select PSBMs based on cellular uptake, subcellular localization, and cytotoxic mechanisms. Perhaps more importantly, these results, demonstrate that biologically informed combinations of PSBMs and free-form photosensitizers may lead to enhanced therapeutic effects. Taken together, these findings suggest that the development of PSBMs should at least include uptake and subcellular localization studies to correlate with and possibly predict PDT efficacy. Further comparison and characterization of the pharmacokinetics and biodistribution of these PSBMs, as well as PDT parameters (e.g., light fluence, drug-light interval) using in vivo or three-dimensional heterocellular models, will be important next steps in the development of PSBMs for anti-cancer PDT.

## Figures and Tables

**Table 1:** Photodynamic therapy (PDT) efficiency of free benzoporphyrin derivative (BPD) and photosensitizing biomolecules (PSBMs) in glioblastoma cells.

	Cell Lines	U251	U87
Groups *		PDT Efficiency **	PDT Efficiency **
Free BPD		1.12% ± 0.00%	1.07% ± 0.00%
16:0 Lyso PC-BPD		2.30% ± 0.03%	2.14% ± 0.29%
DSPE-PEG-BPD		0.29% ± 0.09%	0.09% ± 0.08%
Cet-BPD		0.94% ± 0.28%	0.57% ± 0.24%

* Drug incubation concentration: 0.25 μM BPD equivalent. ** PDT efficiency: percent of viability reduction per fmole of intracellular BPD. PDT drug-light interval: 24 h. PDT light dose: 690 nm, 20 J/cm^2^, 150 mW/cm^2^. 16:0 Lyso PC-BPD: 1-palmitoyl-2-hydroxy-sn-glycero-3-phosphocholine-BPD; DSPE-PEG-BPD: distearoylphosphoethanolamine-polyethylene glycol-BPD; Cet-BPD: cetuximab-BPD.

## Supplementary Materials

The following are available online at https://www.mdpi.com/2077-0383/8/9/1269/s1, Figure S1: MALDI-TOF mass spectrometer analysis of 16:0 Lyso PC-BPD and DSPE-PEG-BPD. (**a**) Purified 16:0 Lyso PC-BPD showed a single primary product with mass of 1196.4 (red line), compared to the multiple products for the crude reaction mixture (blue line) (yield: 20.1% ± 3.8%). (**b**) Purified DSPE-PEG-BPD demonstrated a shift peak mass at 3602.1 (red line) compared to the crude reaction mixture (blue line) (yield: 13.0% ± 3.7%), Figure S2: The purity of Cet-BPD was examined by monitoring free BPD molecules with gel fluorescence imaging analysis following sodium dodecyl sulfate polyacrylamide gel electrophoresis (SDS-PAGE). (**a**) Coomassie blue staining of SDS-PAGE for visualization of the standards, Cet, Cet-BPD, and free BPD. Conjugation of BPD on Cet led to the aggregation of Cet-BPD after linearization with SDS due to hydrophobicity of BPD. (**b**) Gel fluorescence imaging (Em: 690 nm) of SDS-PAGE shows <1% free BPD impurity in Cet-BPD; fluorescence intensity was quantified using ImageJ., Figure S3: Comparison of PSBMs and free BPD uptake in human glioblastoma cell lines. Quantification of (**a**) free BPD, (**b**) 16:0 Lyso PC-BPD, (**c**) DSPE-PEG-BPD, and (**d**) Cet-BPD intracellular BPD concentrations at 24 h post-incubation, in U251 and U87 cells, using extraction method. (*n* = 5, *n.s.*: nonsignificant), Figure S4: Quantification of BPD fluorescence signal in microscopy images of glioblastoma cells at 24 h post-incubation with free BPD, 16:0 Lyso PC-BPD, DESP-PEG-BPD, and Cet-BPD (0.25 µM). Quantification of fluorescence signal as determined by ImageJ software [33] (*n* = 4, * *p* < 0.05, *** *p* < 0.001, *n.s.*: nonsignificant, one-way ANOVA with Tukey’s post hoc test), Figure S5: Apoptosis and necrosis quantification. Analysis showing the mean values of cell subpopulations (Q1: Annexin V−/PI+, Q2: Annexin V+/PI+, Q3: Annexin V+/PI−, Q4: Annexin V−/PI−). (*n* = 5, * *p* < 0.05, *n.s.*: nonsignificant), Figure S6: Analysis showing the mean fluorescent intensity (MFI) of LysoTracker Red staining in U251 cells after PDT with different PSBMs or free BPD. (*n* > 3, *n.s.*: nonsignificant, one-way ANOVA with Tukey’s post hoc test).

## Abbreviations

1-ethyl-3-(3-dimethylaminopropyl) carbodiimide	(EDC)
1-palmitoyl-2-hydroxy-sn-glycero-3-phosphocholine	(16:0 Lyso PC)
4-(dimethylamino) pyridine	(DMAP)
aminolevulinic acid hydrochloride	(ALA HCL)
benzoporphyrin derivative	(BPD)
carbonilcyanide p-triflouromethoxyphenylhydrazone	(FCCP)
cetuximab	(Cet)
dimethyl sulfoxide	(DMSO)
distearoyl-phosphoethanolamine-polyethylene-glycol	(DSPE-PEG)
fluorescence-activated cell sorting	(FACS)
fluorescence intensity	(FI)
matrix-assisted laser desorption ionization-time of flight mass spectrometry	(MALDI-TOF MS)
mean fluorescence intensity	(MFI)
meso-tetrahydroxyphenyl chlorin	(mTHPC)
mitochondrial membrane potential	(ΔΨm)
N,N-diisopropylethylamine	(DIPEA)
N-hydroxysuccinimide	(NHS)
near-infrared	(NIR)
patient-derived xenograft	(PDX)
epidermal growth factor receptor	(EGFR)
phosphate buffered saline	(PBS)
photosensitizing biomolecules	(PSBM)
polyethylene glycol	(PEG)
protoporphyrin IX	(PpIX)
reactive molecular species	(RMS)
singlet oxygen	(^1^O_2_)
singlet oxygen sensor green	(SOSG)
standard error of the mean	(SEM)
sodium dodecyl sulfate-polyacrylamide gel electrophoresis	(SDS-PAGE)
The United States Food and Drug Administration	(FDA)
