# Supplementary material for: Systematic Evaluation of Light-Activatable Biohybrids for Anti-Glioma Photodynamic Therapy
Source: J Clin Med. 2019 Aug 21;8(9):1269. doi: 10.3390/jcm8091269 (PMC6780262; doi:10.3390/jcm8091269)
Supplement: Supplementary file 1 [file jcm-08-01269-s001.pdf]

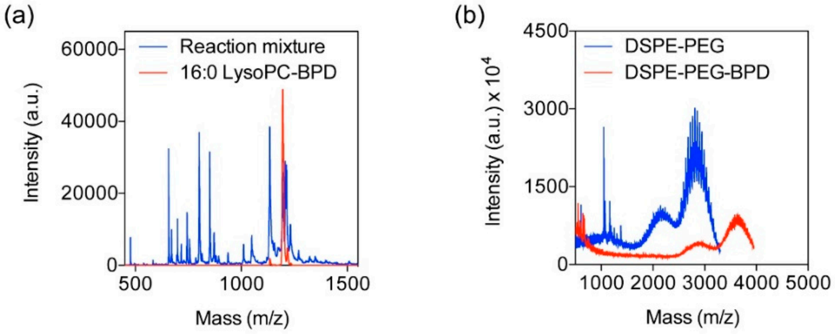

Figure S1. MALDI-TOF mass spectrometer analysis of 16:0 Lyso PC-BPD and DSPE-PEG-BPD. (**a**) Purified 16:0 Lyso PC-BPD showed a single primary product with mass of 1196.4 (red line), compared to the multiple products for the crude reaction mixture (blue line) (yield: 20.1% ± 3.8%). (**b**) Purified DSPE-PEG-BPD demonstrated a shift peak mass at 3602.1 (red line) compared to the crude reaction mixture (blue line) (yield: 13.0% ± 3.7%).

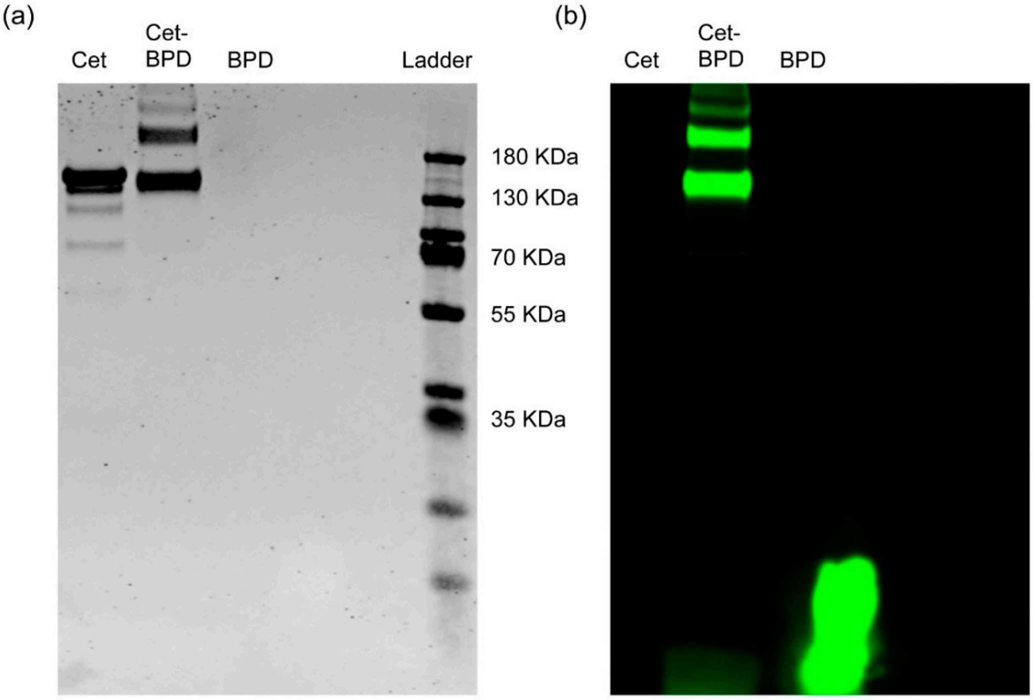

Figure S2. The purity of Cet-BPD was examined by monitoring free BPD molecules with gel fluorescence imaging analysis following sodium dodecyl sulfate polyacrylamide gel electrophoresis (SDS-PAGE). (**a**) Coomassie blue staining of SDS-PAGE for visualization of the standards, Cet, Cet-BPD, and free BPD. Conjugation of BPD on Cet led to the aggregation of Cet-BPD after linearization with SDS due to hydrophobicity of BPD. (**b**) Gel fluorescence imaging (Em: 690 nm) of SDS-PAGE shows <1% free BPD impurity in Cet-BPD; fluorescence intensity was quantified using ImageJ.

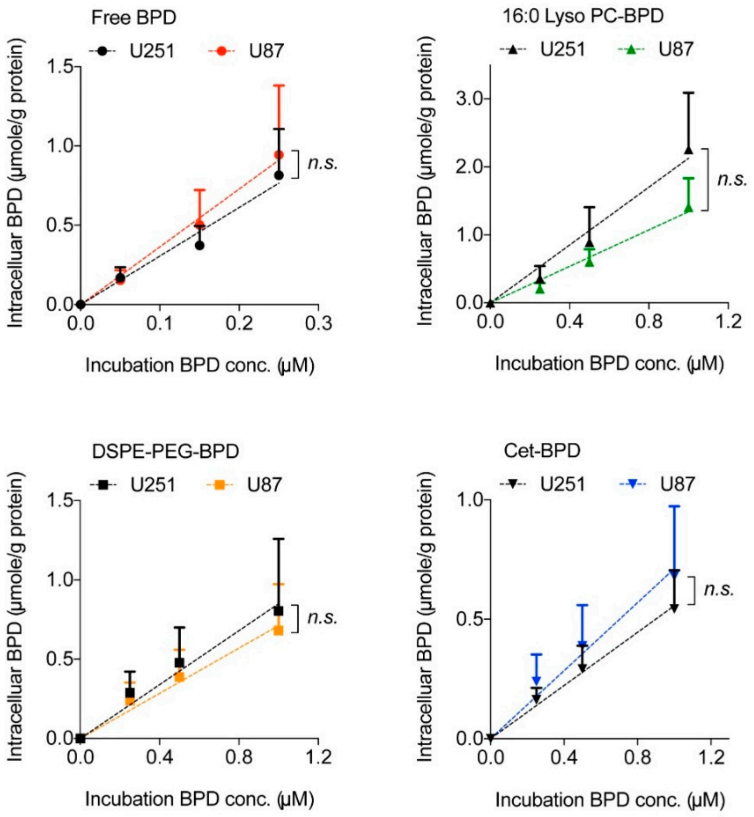

Figure S3. Comparison of PSBMs and free BPD uptake in human glioblastoma cell lines. Quantification of (**a**) free BPD, (**b**) 16:0 Lyso PC-BPD, (**c**) DSPE-PEG-BPD, and (**d**) Cet-BPD intracellular BPD concentrations at 24 h post-incubation, in U251 and U87 cells, using extraction method. (*n* = 5, *n.s.*: nonsignificant).

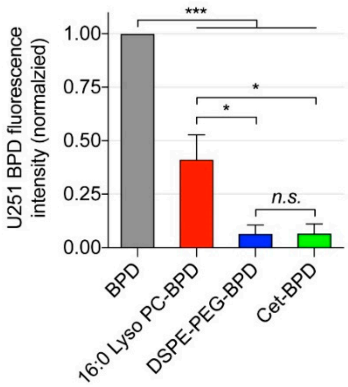

Figure S4. Quantification of BPD fluorescence signal in microscopy images of glioblastoma cells at 24 h post-incubation with free BPD, 16:0 Lyso PC-BPD, DESP-PEG-BPD, and Cet-BPD (0.25 µM). Quantification of fluorescence signal as determined by ImageJ software [33] ($n = 4$, * $p < 0.05$, *** $p < 0.001$, *n.s.*: nonsignificant, one-way ANOVA with Tukey's post hoc test).

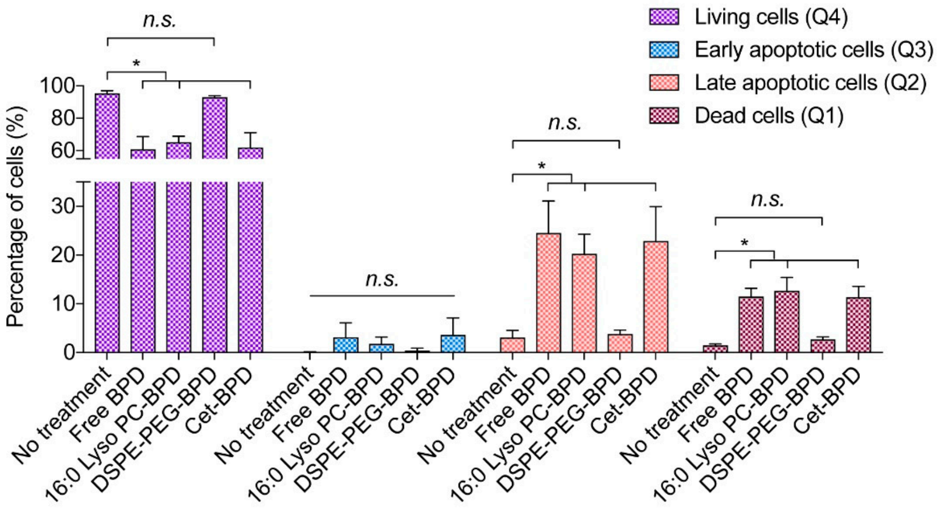

Figure S5. Apoptosis and necrosis quantification. Analysis showing the mean values of cell subpopulations (Q1: Annexin V−/PI+, Q2: Annexin V+/PI+, Q3: Annexin V+/PI−, Q4: Annexin V−/PI−). ($n = 5$, * $p < 0.05$, *n.s.*: nonsignificant).

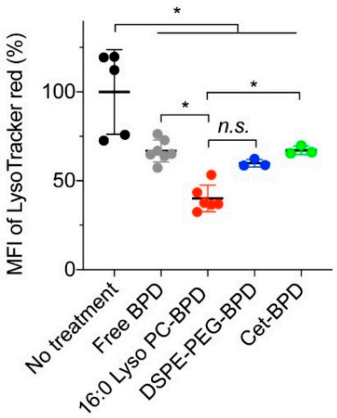

Figure S6. Analysis showing the mean fluorescent intensity (MFI) of LysoTracker Red staining in U251 cells after PDT with different PSBMs or free BPD. ($n > 3$, *n.s.*: nonsignificant, one-way ANOVA with Tukey's post hoc test).
